# Self-Care for Nurses and Midwives: Findings from a Scoping Review

**DOI:** 10.3390/healthcare10122473

**Published:** 2022-12-07

**Authors:** Luisa Sist, Sara Savadori, Annalisa Grandi, Monica Martoni, Elena Baiocchi, Carlotta Lombardo, Lara Colombo

**Affiliations:** 1 IRCCS Azienda Ospedaliero, Universitaria di Bologna, 40138 Bologna, Italy; 2 Italy Midwife—Delivery Room, “M.Bufalini” Hospital, 47521 Cesena, Italy; 3 Department of Psychology, University of Turin, 10124 Turin, Italy; 4 Department of Experimental, Diagnostic and Specialty Medicine, University of Bologna, 40138 Bologna, Italy; 5 Italy Midwife—Delivery Room, “Infermi” Hospital, 47923 Rimini, Italy

**Keywords:** self-care, healthcare professionals, midwives, nurses, mindfulness, resilience, compassion

## Abstract

Self-care for health care professionals is essential in order to optimize the care they provide and to prevent serious consequences for their health. This scoping review aimed to identify (a) the concepts used in the literature to describe self-care; (b) interventions that influence self-care. The scoping review was conducted according to the criteria and methodology by Arksey and O’Malley, from November 2020 to January 2021, by consulting the following databases: Pubmed, CINAHL, Scopus, PsycInfo, Cochrane Library, Joanna Briggs Library. Various keywords and MesH terms were used for the search, including self-care, nurses, midwives, nursing, midwifery, self-compassion, and self-awareness. Eighteen studies were included. The concept of self-care is related to three constructs: (a) Mindfulness; (b) Compassion; and (c) Resilience. In the literature, self-care interventions can be distinguished as (a) mindfulness-based; (b) educational; (c) multimodal approach; and (d) mind-body interventions. In recent years, the concept of self-care is a topic of great interest in the literature; dealing with self-care from both a theoretical and a practical—personal and professional—perspective has become more important in order to promote practitioners’ well-being. This scoping review helps to clarify the terms related to self-care and looks at tested interventions to improve the well-being of caregivers.

## 1. Introduction

The World Health Organization defines self-care as the ability of individuals, families, and communities to promote health, prevent disease, maintain health, and cope with illness and disability, with or without the support of a health professional [1]. The concept refers not only to the patient but also to the caring professions, among them nurses and midwives. Nurses and midwives promote their own health (physical, psychological, social, spiritual, and emotional) through self-care, in order to provide quality care to patients [2].

The concept of self-care originated with the theorist Dorothea E. Orem in 1956 and refers to actions taken by staff to compensate or overcome limitations associated with patients’ health in self-care [3].

The term self-care appears as a concept that has evolved over the years, up to the present day, in which seven components can be identified, referring to: (a) health in the sense of prevention and maintenance of health/life, health promotion; (b) illness or disability, including the treatment of illness or symptoms and the discovery of disease; (c) general outcomes, as self-care, as a continuum of caring, helps individuals to build the capacity to look after themselves; (d) those who perform self-care, which can be understood as a characteristic attributed to the whole population or as care on the part of others; (e) the actions of self-care, which implies the assumption of responsibility; (f) health professionals, as self-care actions can be directed by professionals, without their involvement or in collaboration with them; (g) the health system, insofar as the individual functions as a primary health resource within the health system [4].

The concept of self-care is related to these concepts: self-care agency, self-management, self-monitoring, symptom management, self-management support, and self-efficacy. According to the literature, a healthy individual puts self-care into practice through actions aimed at promoting health and maintaining psychophysical well-being; when an acute or chronic illness occurs, the individual continues to carry out these activities, in order to keep the disease stable (self-care maintenance) by monitoring signs and symptoms (self-monitoring), with the support of health professionals who provide information on treatment and help to develop psychomotor skills (self-management support) or directly manage symptoms and side effects of treatments (disease management) [5].

The concept also appears within a relationship-based theory, which is an evidence-based model that helps to create a healing environment in healthcare organizations. One of its key components is the relationship with oneself as well as with patients, family members, and colleagues. The relationship with oneself is important because it requires a knowledge of oneself and of personal satisfaction factors in order to select effective self-care strategies that increase resilience [6]. 

During this period of the pandemic caused by the SARS-CoV-2 virus, Coronavirus disease (COVID-19) (defined according to the World Health Organization classification) has had effects on the population, not only in terms of illness, but also in terms of mental-psychological disorders, such as emotional exhaustion at work [7], depression [7,8], anxiety, mood disorders, psychological distress, post-traumatic stress disorder, insomnia, fear, stigmatization, low self-esteem, lack of self-control, and other adverse mental health outcomes [8].

The existing literature has shown that healthcare professionals engaged in the frontline of the COVID-19 disease have health outcomes such as increased stress in the workplace, increased sleep disturbance [9,10], the risk of developing an anxiety disorder [11], and depression [10].

Organizations need to understand the root causes of distress so that they can treat patients by providing efficient and safe care [12]. In order to continue to provide efficient and safe care for patients, healthcare professionals must address their own mental health [13] by using prevention and health-promotion mechanisms [14]. In the literature, as described above, next to the concept of care appears the concept of self-care, referring to care toward the professional. The concept is linked to caring for others [15], as an essential element in optimising care [16].

Within this framework, there is a need to study the self-care concept in order to foster the development of self-care-based programs or training for health care professionals, both in general and, in particular, during this period of the COVID-19 pandemic [12]. The aim of the present work is to identify recent evidence of self-care, specifically in reference to nurses and midwives. The main objective of the study was to summarize the concept of self-care and the interventions that influence it described in the literature. The two specific questions we used to guide this review were, most importantly, (1) identifying and summarizing the concept of self-care; and, as a supplementary focus, (2) identifying interventions that promote self-care.

## 2. Materials and Methods

A scoping review was conducted on the basis of a methodological framework proposed by Arksey and O’Malley [17], involving the following steps: (a) identification of the research question; (b) identification; (c) selection; (d) collection, synthesis and reporting of the studies. The search protocol had not been previously registered. To facilitate the electronic search, the search questions were structured according to the PICO model [number to be inserted]: (1) P (population): nurses and midwives, I (intervention): any intervention, C (comparison intervention): no comparison, O (clinical outcome): self-care concept; (2) P (population): nurses and midwives, I (intervention): self-care interventions, C (comparison intervention): no self-care intervention, O (clinical outcome) self-care skills.

The scoping review was carried out between November 2020 and January 2021 by consulting electronic databases: Pubmed, Cumulative Index to Nursing and Allied Health Literature (CINAHL), Scopus, PsycInfo, Cochrane Library, Joanna Briggs Library. The keywords used for the literature search were: self-care, nurses, midwives, nursing, midwifery, self-compassion, and self-awareness. The following Medical Subject Heading/Emtree terms and free terms were also used: self-care, Self-Care/psychology nurses, midwives, nurses, nursing, midwifery. There are no terms in the review to define self-care interventions because it is a secondary objective. The inclusion criteria were:

(a) all primary studies that had available abstracts;
(b) articles published within the past 10 years, in order to find current sources;
(c) articles published in English or Italian.

Two authors (SS, LS) independently reviewed the titles and abstracts of the articles identified in the research based on the inclusion criteria and decided which documents should be read. Articles were excluded at this stage if the title or abstract did not focus on our topic, were not in English, or were not original research. In the event of disagreement, a third author (AG) of the review was consulted. The selection process was summarized with the PRISMA-ScR methodology [18]. The authors independently synthesized the studies included by listing the following elements: reference, regions/clinical working area, study design, objective, sample, findings.

A third author (AG) was consulted when divergent opinions emerged regarding clinical evaluation, and these were then discussed within the research team and consensus was obtained. The quality was assessed using the Standard Quality Assessment Criteria tool to evaluate the quality of the quantitative and qualitative studies. Each statement received the score of either 0 points (not addressed), 1 point (partially addressed), or 2 points (fully addressed). The total scores were reported as a percentage using the method of Kmet and colleagues. All of the studies considered in this review obtained a score of above 60, a limit defined by the authors of the instrument as reasonable. The studies were considered and scored according to the Checklist of Criteria for Quality Assessment of Quantitative Studies [19] and had a summary score of above 66.6% for quantitative studies and 80% for qualitative studies (Table 1).

## 3. Results

**Figure 1:**
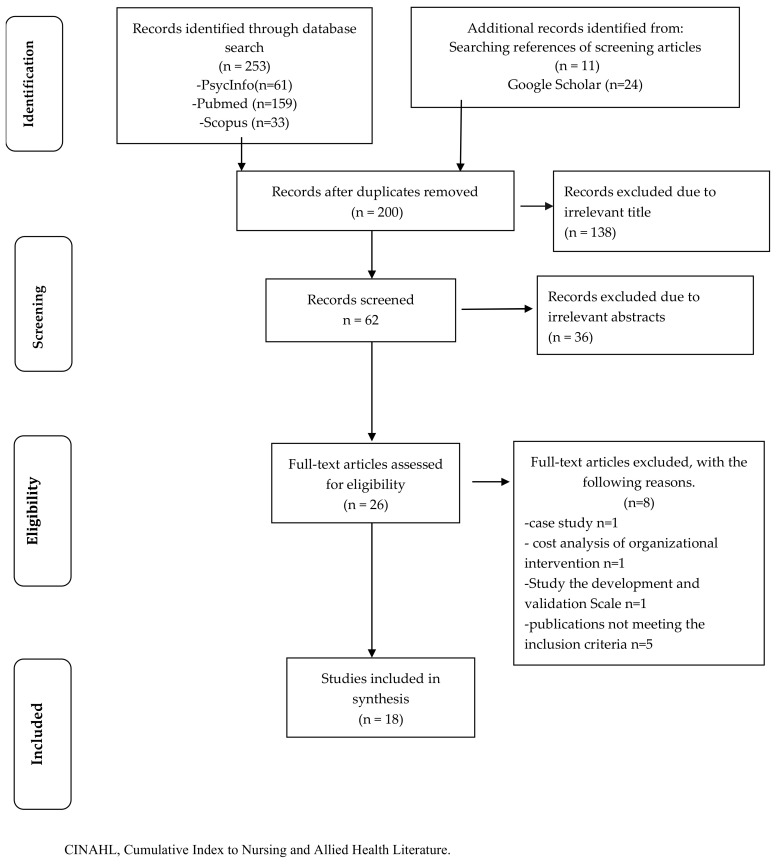
Flow chart illustrating the selection process of scoping review [18].

The online searches yielded 253 articles, and an additional 35 similar articles were identified from other sources (e.g., searching references of screening articles and records identified with Google Scholar). After the removal of duplicates, a total of 200 single articles emerged; following this evaluation, 62 titles were selected; subsequently, after reading the abstracts, 26 were selected for full-text screening. After reading the abstracts, 18 studies were identified as eligible (Figure 1) and synthesized according to the data extraction process (Table 2).

### 3.1. The Terms of the Concept and Conceptual Frameworks

Overall, 14 studies [20,21,22,23,24,25,26,27,28,29,30,31,32,33] were included that describe the concept; four of these are qualitative studies [20,31,32,33] and three are cross-sectional [24,26,28]. The authors also considered seven pre-post-intervention studies [21,22,23,25,27,29,30] for the description of the concept, as they define some key concepts.

Self-care means activating a process of measures and activities to be developed in order to stabilize and maintain health [20]. The responsibility for this process is both of the individual, who should apply the methods regularly, and of the institution, which must offer programs that meet the needs of the staff [21]. Two other terms appear in the literature, in addition to the term self-care: self-care ability, understood as the ability to take care of oneself [27], and self-care strategies, understood as the strategies adopted to promote one’s holistic well-being [28], e.g., developing a positive attitude, recognizing one’s uniqueness, and one’s contribution to work [32]. Self-care is influenced by the vision of nursing identity; nurses need permission from the environment in which they work and from themselves to care for themselves and to be self-compassionate. Nurses need to be supported in the process of self-care and self-compassion in order to gain the skills to manage their own emotions, in dealing with patients’ needs and, consequently, to prevent negative effects such as burnout and compassion fatigue [20]. The effects of self-care are that it builds resilience and decreases burnout and secondary trauma in healthcare workers [21]. Within the broad concept of the process of taking charge of oneself, three other terms related to this concept appear in the literature: (a) Mindfulness, (b) Compassion, and (c) Resilience.

Mindfulness is defined as a self-care practice [25] that involves: (a) knowing how to move toward discomfort or pain from an awareness of negative thoughts and feelings [23]; (b) mindfulness is defined as awareness of the moment that emerges through paying attention on purpose, in the present moment, to allow the person to effectively detach (de-center) from experiences, thus facilitating a more balanced [22] and flexible response [30]. Alongside the concept of mindfulness appears the concept of self-awareness, which is related to an awareness or consciousness of one’s reactions [33]. Other terms have been found in the literature alongside the term mindfulness: (a) dispositional mindfulness, or the ability “to be mindful” [29]. The literature supports the idea that mindfulness has protective effects against stressors at home and at work [27].

Compassion can also be defined as the ability and desire to be present in another person’s moments of despair [33]. The factors that the term includes are basic kindness, mindfulness, humanity [23], and empathy, i.e., when observing another person’s affectivity [24]. With the term compassion we find:

(a) self-compassion means directing compassion towards oneself with kindness and is therefore important for self-care [28]. It is composed of three factors: self-kindness, a sense of common humanity, and mindfulness [23]. Self-compassion involves being touched by one’s own suffering, generating the desire to care with kindness [24] and gives one the ability to hold back one’s feelings of suffering without reacting with self-criticism [23]; 

(b) satisfaction with compassion is defined as the positive aspects of one’s professional quality of life, e.g., positive feelings regarding one’s role as a caregiver [20,22]. This allows caregivers to engage in meaningful interactions with patients [24] and is a positive indicator of personal coping resources in caregivers [22].

Actions to soothe [23] are not specific nursing actions [20,33], but are defined as compassionate care in reference to human interactions. Compassion can be a protective factor in psychological well-being as it provides a self-other distinction that is essential to regulate feelings of personal distress [24]. By cultivating resilience, or, specifically, “the five antibodies” (self-regulation, intentionality, perceptual maturation, social connection and support, self-care and revitalization), it is possible to avoid incurring compassion fatigue, a negative psychological outcome related to compassion [22]. Compassion fatigue is an acute syndrome characterized by the combination of Secondary Traumatic Stress (STS) and burnout due to the demands of caring for individuals in need [22]. Compassion fatigue involves a state of reduced capacity for compassion due to being exhausted from dealing with the suffering of others [24]. Compassion fatigue is characterized by a lack of balance between compassion satisfaction and quality of work life, as well as feelings of hopelessness [21]. The effects of this work-related trauma typically occur in the form of secondary exposure through working with patients who have experienced or are experiencing trauma, resulting in difficulty sleeping, intrusive imagery, and the avoidance of memories of traumatic experiences [26].

Resilience is a multidimensional construct and involves ways of thinking and behaving that can be learned [30]. It is defined as the ability to withstand significant disruption and change through effective coping and the capacity to undergo personal change that allows for personal growth [21].

The concept of resilience has as a construct self-efficacy, defined as a person’s belief that they can effectively perform a given task [26]. Strategies are identified to promote coping and resilience, such as facilitating social connections, promoting positivity, capitalizing on nurses’ strengths, nurturing nurses’ growth, encouraging nurses’ self-care, fostering mindfulness practice, and conveying altruism [31].

### 3.2. Interventions Associated with Nurses/Midwives’ Self-Care

To answer the question regarding interventions associated with nurses/midwives’ self-care, 11 studies [21,22,23,25,27,29,30,34,35,36,37] were selected and summarized with intervention, outcome, assessment tool, which are presented in Table 3. The self-care interventions are:

(a) Caring for the caregivers (CCG)

This is a long-term multimodal intervention in which a series of combined self-care practices are provided, combined with mindfulness, and in which self-awareness is encouraged. The five components included in the intervention are: Cognitive (mindfulness), Somatic (relaxation), Emotional/Expressive (drawing/journaling/listening circle), Dynamic-Interactive (movement/interactive dance), and Hands-on (acupressure/shiatsu). The aim of the intervention is to provide manageable and useful self-care tools to prevent stress [29].

(b) The PERMA model is a long-term educational intervention that is based on positive psychology workshops, which comprise a series of voluntary activities that address five components of the model: Positive Emotions (P), Attraction (E), Positive Relationships (R), Meaning (M), and Achievement (A). The objective of this intervention is to promote people’s well-being, which has a great impact on workforce retention and the quantity and quality of services offered [37].

(c) Mind-body practices and yoga instruction

This is a mind-body practices intervention in which yoga is practiced for three weeks.

The aim is to improve self-awareness, helping individuals to become more aware in daily activities and functions and improving their health and well-being [34].

(d) Mindful Self-Compassion (MSC) training intervention

This is an eight week training intervention in mindfulness meditation techniques, in which the fundamental principles and practices of MSC are taught, including Mindfulness Meditation (MM), Loving Kindness Meditation (LKM) and Compassion Meditation (CM). The aim of this program is to help participants develop self-compassion, with a secondary emphasis on mindfulness [23].

(e) Mindful self-care and resilience (MSCR) intervention

This is a four week training intervention in mindfulness meditation techniques that includes educational workshops on compassion fatigue and resilience, followed by a series of weekly workshops on mindfulness skills. The focus is on learning mindfulness to support resilience skills and mindfulness by promoting value-based actions and self-care. [22,30].

(f) Mindfulness Based Stress Reduction (MBSR)

This is an intervention that includes practical mindfulness training techniques with an emphasis on self-compassion training over an eight week period. It aims to provide practical strategies through mindfulness practice on a daily basis to improve physical and psychological well-being and to promote self-care needs [25,27].

(g) Organizational intervention

These are interventions that support staff through organizational tools and support. The support interventions consist of posters promoting self-care, team-bonding sessions and end-of-shift meetings; a pocket card and posters promoting self-care and resilience; team bonding sessions; Recognize and Reflect, a short discussion group focused on guided work; and an end-of-shift staff meeting. The aim is to support staff through mutual support and experience sharing [36].

(h) Six resilience workshops and a mentoring program

This intervention consists of six resilience workshops and a mentoring program, conducted over a period of six months. The modalities used are group discussion and individual, pair, and group learning activities. The workshop was developed through the following themes: positive relationships and networks, mentoring, positive outlook, resilience, intellectual flexibility, emotional intelligence, life balance, spirituality, reflection and critical thinking. The aim of the program was to promote strategies that can reduce work-related stress [35].

(i) THRIVE© program

This is an eight hour educational intervention designed to teach self-care strategies, followed by a six week private group study on a social media platform and a final two hour session. The program also includes mindfulness sessions, art, journaling, guided imagery, and acupressure. The aim is to increase the quality of the nurses’ work by teaching self-care activities that are used in the daily routine [21].

**Table 1:** Critical appraisal of reviewed studies.

Quantitative Studies	Alexander et al. [34] 2015.	Blackburn et al. [21] 2020.	Craigie et al. [22] 2016.	Delaney [23] 2018.	Duarte et al. [24] 2016.	Fourer et al. [25] 2013.	Hegney et al. [26] 2015.	Mahon et al. [27] 2017.	Mills et al. [28] 2018.	O’Riordan et al. [36] 2020.	Sallon et al. [29] 2015.	Shaghaghi et al. [37] 2021.
1 Question/objective sufficiently described?	Y	P	Y	Y	Y	P	Y	Y	Y	Y	Y	Y
2 Study design evident and appropriate?	Y	P	Y	Y	Y	Y	Y	Y	Y	Y	Y	Y
3 Method of subject/comparison group selection or source of information/input variables described and appropriate?	Y	P	Y	Y	P	Y	Y	Y	Y	P	Y	Y
4 Subject (and comparison group, if applicable) characteristics sufficiently described?	Y	P	Y	Y	P	N	Y	Y	Y	P	Y	Y
5 If interventional and random allocation was possible, was it described?	P	N/A	N/A	N/A	N/A	N/A	N/A	N/A	N/A	N/A	N/A	Y
6 If interventional and blinding of investigators was possible, was it reported?	N	N/A	N/A	N/A	N/A	N/A	N/A	N/A	N/A	N/A	N/A	N
7 If interventional and blinding of subjects was possible, was it reported?	N	N/A	N/A	N/A	N/A	N/A	N/A	N/A	N/A	N/A	N/A	N
8 Outcome and (if applicable) exposure measure(s) well defined and robust to measurement/misclassification bias? Means of assessment reported?	Y	Y	Y	Y	Y	Y	Y	Y	Y	Y	Y	Y
9 Sample size appropriate?	Y	N/A	N/A	Y	N/A	N/A	N/A	N/A	P	N/A	N/A	N/A
10 Analytic methods described/justified and appropriate?	Y	P	Y	Y	Y	P	Y	Y	Y	Y	Y	Y
11 Some estimate of variance is reported for the main results?	P	N	Y	Y	Y	P	Y	Y	P	Y	P	Y
12 Controlled for confounding?	N	N/A	N/A	N/A	N/A	N/A	N/A	N/A	N/A	N/A	N/A	N/A
13 Results reported in sufficient detail?	Y	Y	Y	Y	Y	P	Y	Y	Y	Y	Y	Y
14 Conclusions supported by the results?	Y	Y	Y	Y	Y	Y	Y	Y	Y	Y	Y	Y
Score (%)	20/28 (71.4)	11/18 (61.1)	18/18 (100)	20/20 (100)	16/18 (88.8)	12/20 (66.6)	18/18 (100)	18/18 (100)	18/20 (90)	16/18 (90)	17/18 (94.4)	24/28 (85.7)
**Qualitative Studies**	**Andrews et al. [20] 2020.**		**Mc Donald et al. [35] 2013.**		**Slatyer et al. [30] 2018.**		**Wei et al. [31] 2019.**		**Wei et al. [32] 2020.**		**Wiklund et al. [33] 2013.**	
1 Question/objective sufficiently described?	Y		Y		Y		Y		Y		Y	
2 Study design evident and appropriate?	Y		Y		Y		Y		Y		Y	
3 Context for the study clear?	Y		Y		Y		Y		Y		Y	
4 Connection to a theoretical framework/wider body of knowledge?	Y		Y		Y		Y		Y		Y	
5 Sampling strategy described, relevant and justified?	Y		P		P		Y		Y		Y	
6 Data collection methods clearly described and systematic?	Y		P		P		Y		Y		Y	
7 Data analysis clearly described and systematic?	Y		Y		Y		Y		Y		Y	
8 Use of verification procedure(s) to establish credibility?	Y		N		N		Y		Y		N	
9 Conclusions supported by the results?	Y		Y		Y		Y		Y		Y	
10 Reflexivity of the account?	Y		Y		Y		0		Y		Y	
Score (%)	20/20 (100)		16/20 (80)		16/20 (80)		18/20 (90)		20/20 (100)		18/20 (90)	

YES (2) = Y PARTIAL (1) = P NO (0) = N N/A = not applicable.

**Table 2:** Characteristics and main results of the studies included in the systematic review.

References	Regions/ Clinical Working Area	Design	Objective	Sample	Findings
Alexander et al. [34] 2015.	An urban 560-bed teaching hospital, Texas Christian University	Pilot Level randomized controlled trial	To examine the efficacy of yoga in improving self-care and reducing burnout among nurses practicing at an urban, tax-supported health care network.	40 nurses	The yoga group showed significant improvement in scores from pre-to post-intervention for self-care (*p* < 0.001), mindfulness (*p* = 0.028), emotional exhaustion (*p* = 0.008), and depersonalization (*p* = 0.007).
Andrews et al. [20] 2020	Norway, hospital	Qualitative A constructivist grounded theory approach	To explore nurses’ experiences of self-care and self-compassion, while looking at how this related to compassionate care giving.	30 nurses	Three concepts were derived from the data: (1) ‘hardwired to be caregivers’ –vocation versus role, (2) needing a stable base, (3) managing the emotions of caring. All three concepts linked to a core process: needing permission to self-care and be self-compassionate. Nurses needed permission from others and from themselves to be self-caring and self-compassionate.
Blackburn et al. [21] 2020	The Arthur G. James Cancer Hospital and Richard J. Solove Research Institute in Columbus, Ohio.	Study pre-post	To develop an evidence-based program to address the concerns of burnout and secondary trauma building on the concept of resilience in oncology staff.	164 oncology staff, of whom 160 were nurses or advance practice nurses	The self-assessments carried out before and after the THRIVE program showed an increase in resilience and a decrease in burnout. The average pre- to post program assessment scores for all groups combined demonstrate a greater than 10-point rise in the resilience scores (from 72 to 85), and an even greater decrease in burnout (from 41 to 23) and secondary trauma scores (from 32 to 19)
Craigie et al. [22] 2016.	Western Australia, Large teaching hospital	Pilot Study Pre-post	To evaluate the feasibility of a mindfulness-based intervention aimed at reducing compassion fatigue and improving emotional well-being in nurses.	21 nurses	The outcomes observed after the intervention were significant improvements in compassion satisfaction, negative affect, obsession passion and burnout reduction; no significant changes were observed for general resilience, anxiety, or traumatic stress.
Delaney [23] 2018.	Aberdeen, Hospital	Pilot study Pre-post Quali-quantitative	To examine the effects of a pilot mindful self-compassion (MSC) intervention; these were assessed by analyzing pre- and post-change scores in self-compassion, mindfulness, secondary trauma burnout, compassion satisfaction, and resilience.	13 nurses.	The Pre- to Post- scores of secondary trauma and burnout declined significantly and were negatively associated with self-compassion (r = −0.62, *p* = 0.02) (r = −0.55, *p* = 0.05) and mindfulness (r = −0.54, *p* = 0.05) (r = −0.60, *p* = 0.03), respectively. Resilience and satisfaction with compassion increased.
Duarte et al. [24] 2016.	Portugal’s north and central region, 4 public hospitals	Cross-sectional	To explore how empathy and self-compassion related to professional quality of life (compassion satisfaction, compassion fatigue and burnout). In addition, we wanted to test whether self-compassion may be a protective factor for the impact of empathy on compassion fatigue	280 registered nurses	Correlations and regression analyses showed that empathy and self-compassion predicted the three aspects of quality of professional life. Empathic concern was positively associated with compassion satisfaction and compassion fatigue. High levels of affective empathy may be a risk factor for compassion fatigue, whereas self-compassion may be protective.
Fourer et al. [25] 2013.	Australia, two metropolitan teaching hospitals	Pilot Study Pre-post Quali-quantitative	The study evaluated the effectiveness of a mindfulness-based stress reduction intervention tailored to the psychological well-being of the nurses and midwives.	20 midwives and 20 nurses	Results included significant improvements in score for the general health questionnaire (GHQ-12); sense of coherence (SOC)—life orientation and the depression, anxiety and stress scale (DASS).
Hegney et al. [26] 2015.	Australia, Queensland Nursing Union	Cross-sectional	To determine the contribution of negative trait and individual psychological resilience in explaining nurses’ quality of professional life.	1743 nurses in the public and private sector and in elderly care	Results showed positive relationships between anxiety, depression and stress, negative affect, burnout, and secondary stress (compassion fatigue). Resilience was confirmed to be a mediator of the relationship between TNA (trait negative affect) and CS (Compassion Satisfaction).
Mahon et al. [27] 2017.	Ireland, 3 university hospital	A quasi-experimental pre-test/post-test	To determine whether a mindfulness meditation and self-compassion training intervention had an effect on nurses’ self-reported levels of perceived stress (PS) and levels of compassion	64 nurses.	The results show that after intervention, levels of perceived stress were reduced and compassion scores increased.
Mc Donald et al. [35] 2013.	Australia women’s and children’s health service, tertiary hospital	Qualitative instrumental collective case study	To evaluate a work-based educational intervention to promote personal resilience.	14 nurses and midwives.	The results of the intervention were a benefit to participants in personal and professional areas and increased confidence, self-awareness, assertiveness, and self-care.
Mills et al. [28] 2018.	Australia, palliative care services	Cross-sectional survey	Examined the levels of, and relationships between, self-care, self-compassion and compassion among those who work in palliative care.	309 nurses and physicians	Levels of compassion, self-compassion, and self-care skills varied. Linear regression also indicated that: (1) an increase in compassion was associated with a decrease in self-compassion; and (2) an increase in self-care capacity was associated with a decrease in self-compassion. The ability to self-care was associated with an increase in self-compassion.
O’Riordan et al. [36] 2020.	Cork University Maternity Hospital, large tertiary hospital	Pilot study Pre-post	To investigate whether an intervention which increases support for staff is feasible to implement and effective at improving staff wellbeing.	28 doctors in training and 69 midwives	The results show that there was a statistically significant decrease in the Burnout score of the quality of professional life before and after.
Sallon et al. [29] 2015.	Israel, Hadassah Medical Organization	Quasi-experimental pre–post study	To evaluate the Caring for the Caregivers intervention, a multimodal approach to stress reduction designed to address the multidimensional nature of stress in hospital staff; it integrates five components: Cognitive, Somatic, Dynamic, Emotional, and Practical, in a flexible eight-month format.	118 participants and 97 controls, 75% were nurses	The results show significant improvements for participants compared to controls in pre-post scores for the Maslach Burnout Inventory, Job-Related Tension Index, Perceived Stress, Productivity Scale, General Health Questionnaire, Positive and Negative Affect Schedule, and visual analogue scales.
Shaghaghi et al. [37] 2021	Iran, community health centers of Mashhad City	Randomized clinical trial with pretest-post-test design with a control group	To investigate the effect of positive psychological interventions on the psychological well-being of midwives.	60 midwives; 30 experiment and 30 control	The results show that there is a significant difference between the post-test scores of the two intervention groups and the control groups in the variables of self-report, and that this program improves the psychological well-being of midwives.
Slatyer et al. [30] 2018	Australia, tertiary acute care hospital	Mixed method Pilot intervention of Mindful Self-Care and Resiliency	To evaluate the effects of a short mindful self-care and resilience (MSCR) program specifically aimed at reducing compassion fatigue and improving nurses’ resilience	16 nurses completed the program.	Participants’ perceptions of the program revealed five themes: gaining perspective and insight; developing feelings of inner calm; taking time for oneself; feasibility and acceptability for oneself; feasibility and acceptability of the program and the use of self-care strategies.
Wei et al. [31] 2019	United States, tertiary hospital	Qualitative descriptive study	To describe strategies of nurse leaders to cultivate nurse resilience. Resilience strategies	20 nurse leaders.	Seven strategies were identified to cultivate nurse resilience: facilitating social connections, promoting positivity, capitalizing on nurses’ strengths, nurturing nurses’ growth, encouraging nurses’ self-care, promoting mindfulness practices and conveying altruism.
Wei et al. [32] 2020	USA, children’s hospital, Pediatric Critical Care Nurses and Physicians	Qualitative descriptive study	To determine the perceptions of self-care strategies to combat professional burnout among nurses and physicians in pediatric critical care settings. Self-care strategies	20 nurses and physicians	The main self-care strategies are: finding meaning in work, connecting with a source of energy, nurturing interpersonal connections, developing an attitude of positivity, performing emotional hygiene, and recognizing one’s uniqueness and contributions to work.
Wiklund et al. [33] 2013.	clinical nursing teachers Sweden	Qualitative phenomenological and hermeneutic interpretation	To explore the understanding of self-compassion as a source of compassionate care. Self-Compassion Watson’s Theory of Human Caring, focus on both compassion for the patients and the caregiver’s ability to self-care Watson’s Caritas Processes 2008	The sample consists of four clinical nursing teachers who met for a total of 12 h of experiential work and reflection.	The results that emerged can be categorized into five themes: being present, with oneself and with others; respect for human vulnerability; being non-judgmental; giving a voice to things that need to be said and heard; and being able to accept the gift of compassion from others.

In Table 3, we presented both the outcomes measured by the intervention as well as the instruments used.

**Table 3:** Synthesis of interventions.

Interventions	Outcome	Assessment Tool	Reference
Caring for the caregivers (CCG)	Burnout	Maslach Burnout Inventory (MBI)	Sallon et al. [29] 2015.
	Individual experiences Positive affect Negative affect	The Positive and Negative Affect Schedule (PANAS)	
	Job tension	Job-Related Tension Index (JRTI)	
	Self-perceived performance	Productivity Scale (PS)	
	Symptom-focused outcome measures	Visual Analogue Scales (VAS)	
	Stress	Perceived Stress Scale (PSS)	
PERMA model	Psychological Well-being	Ryff’s Psychological Well-Being questionnaire	Shaghaghi et al. [37] 2021.
Mind-body practices Yoga instruction	Self-care	Health Promoting Lifestyle	Alexander et al. [34] 2015.
		Profile II (HPLP II)	
		Mindfulness Inventory (FMI)	
	Burnout	Maslach Burnout Inventory (MBI)	
Mindful Self-Compassion (MSC) training intervention	Compassion Satisfaction	The Professional Quality of Life Scale version 5 (ProQoL5)	Delaney [23] 2018.
	Self-compassion:	The Neff 26-item Self-compassion scale	
	Mindfulness	The Freiburg Mindfulness inventory,	
	Resilience	Connor-Davidson Resilience Scale (CD-RISC 25)	
	Burnout	The Professional Quality of Life Scale version 5 (ProQoL5)	
	Secondary Trauma	The Professional Quality of Life Scale version 5 (ProQoL5)	
Mindful selfcare and resiliency (MSCR) intervention	Compassion Fatigue Compassion satisfaction	The Professional Quality of Life Scale version 5 (ProQoL5	Craigie et al. [22] 2016.
	Mindfulness	Individual, unstructured interview	
	Resilience	Connor-Davidson Resilience Scale (CD-RISC)	
	Depression, anxiety, and stress symptoms	Depression Anxiety Stress Scales (DASS)	
	Harmonious and obsessive passion	Passion for Work Scale (PWS)	
	Trait negative affect	Spielberger State-Trait Anxiety Inventory form Y2 (STAIY2)	Slatyer et al. [30] 2018.
Mindfulness Based Stress Reduction (MBSR)	Compassion	Compassion Scale (CS).	Mahon et al. [27] 2017.
	Stress	Perceived Stress Scale (PSS)	
	Depression, anxiety and stress symptoms	Depression Anxiety Stress Scales (DASS)	Fourer et al. [25] 2013.
	Self-perceived general health	The SOC—Orientation to Life	
Organizational Intervention	Compassion Fatigue	The Professional Quality of Life Scale version 5 (ProQoL5)	O’Riordan et al. [36] 2020.
	Burnout	Maslach Burnout Inventory (MBI)	
Six resilience workshops and a mentoring program	Resilience	Face-to-face, semi- structured interviews	Mc Donald et al. [35] 2013.
THRIVE© program	Resilience	Connor-Davidson Resilience Scale (CD-RISC)	Blackburn et al. [21] 2020.
	Burnout	Compassion Fatigue Short Scale (CFSS)	
	Secondary Trauma	Compassion Fatigue Short Scale (CFSS)	

## 4. Discussion

In recent years, the concept of self-care has been a topic of great interest in the literature. Dealing with self-care, both from a theoretical and practical perspective, and from a personal and professional point of view, has become increasingly important in order to promote the well-being of practitioners [38]. The scoping review summarised 18 studies concerning the concept of self-care and the interventions associated with nurses/midwives’ self-care.

### 4.1. The Findings about the Concept of Self-Care

In terms of the first question, regarding the meaning of self-care for nurses and midwives, the definition that emerged from the literature reflects a shift in focus from the concept of self-care itself, i.e., taking care of oneself, to an umbrella term for all aspects related to how to take care of oneself [12]. Terms that are already familiar within the literature are used, such as mindfulness, compassion, and resilience, and are linked to the general term that is ‘self-care’ [39].

An example that encapsulates these meanings, but more importantly demonstrates that the variables are positively associated with each other, is provided by Garcia and authors’ study investigating the impact of the COVID-19 pandemic on palliative care workers. The study showed that staff with greater resilience and those who had been working for a longer period reported high levels of self-care and compassion, as they implemented interventions that promoted self-care, mindfulness, and compassion. In addition, he claims that staff who practise mindful self-care behaviours are at a lower risk of developing burnout [40].

The terms used to date are mindfulness, compassion, and resilience, related to the general term of self-care. The terms have different meanings: mindfulness is a practice of self-care [22], compassion is the ability to be present with oneself and others and to accept the compassion of others [33], and resilience is the ability to withstand change through coping [35]. The results that emerged from the concepts of self-care, mindfulness, compassion, and resilience indicate that self-efficacy is present for each individual; it is an individual ability that is presented together with a positive attitude, flexibility, and critical thinking [26], with the intention [23,33] of taking care of oneself and others [23,24,28].

A recent concept analysis emphasised the definition of self-care as the ability to take care of oneself through awareness, self-control, and self-confidence in order to achieve, maintain and promote optimal well-being and health [39]. With the knowledge of this definition, one can identify possible effective strategies to support the active process that people undergo to engage effectively in their own care [41]. Therefore, self-care is a continuous decision-making process that involves becoming aware of one’s emotional states, beliefs, and preconceived ideas [26,41].

### 4.2. The Findings Regarding Interventions Associated with Nurses/Midwives’ Self-Care

The scoping review highlights strategies to promote self-care by proposing different types of interventions, such as those that are mindfulness-based [22,23,25,27,30], educational [35,37], multimodal approach [21,29], and mind-body-based [28,34].

The interventions appear to be structured, individualised, with team modalities, long-term [21,22,23,25,27,29,30,34,35,37] and organisational interventions [36]. From the scoping review, only one study that included organisational interventions was found. The difficulty of conducting organisation-focused interventions emerges, both due to the (lack of) participation of staff and because the modality of team sessions is not always applicable in the long term [36]. Each self-care promotion intervention is measured by the following outcomes, referring to the construct of self-care:

(a) Self-care per intervention Mind-body practices yoga instruction [34];
(b) Mindfulness for Mindful Self-Compassion (MSC) training intervention [23,30];
(c) Compassion for Mindful Self-Compassion (MSC) training intervention [23], Mindfulness Based Stress Reduction (MBSR) [27], Mindful self-care and resiliency (MSCR) intervention [22], and Organisational Intervention [36];
(d) Resilience for Mindful self-care and resiliency (MSCR) intervention [22], Mindful Self-Compassion (MSC) training intervention [23], THRIVE© program [21], and six resilience workshops and mentoring program [35].

The interventions described within this scoping review appear to be viable strategies within the contexts of promoting and improving physical and psychological well-being [25,27,34,37]; to provide tools and actions based on values and self-care [21,22,25,27,29,30]; to improve self-awareness [34], and self-compassion [23], thus preventing negative outcomes such as burnout [21,23,29,34,36], depression, anxiety, and stress symptoms [22,25], harmonious and obsessive passion [22]; job tension [29]; individual experiences positive affect negative affect [29]; psychological well-being [37]¸ secondary Trauma [21,23]; self-perceived general health [25,29]; symptom-focused outcome measures [29]; stress [27,29]; trait negative affect [22].

Our results are in line with a recent review that highlights the importance of the work context in promoting workplace well-being. Interventions, particularly mindfulness-based programs, appear to be beneficial in a supportive environment (e.g., creating favourable conditions, such as protected times and spaces, to engage in self-care).

Mindfulness interventions must be carried out over a long period of time, as mindfulness is a skill that is acquired over time and requires intensive training. In the literature, according to a systematic review, interventions are behavioural and/or educational, with the aim of improving one of the risk factors for individual health (overweight, obesity, smoking habits), clinical (heart disease, stroke, cancer, hypertension) and psychological (work-related stress, mood). Furthermore, the review emphasises that all of these interventions are effective in all dimensions of the person, both individually and in combination. In a particularly complex and changing work environment, interventions that are not only aimed at the individual person, but also at the organisational level, are necessary [42].

This is a real paradigm shift that involves both the individual and the organisation. More specifically, it is important that during the course of the intervention, professionals should feel safe in engaging in self-care at work and in sharing difficulties with colleagues [43].

At this timepoint in the COVID-19 epidemic, it is important to pay attention to the health of healthcare professionals, particularly by providing psychological support [10] or resilience, coping, and self-efficacy programs or models of targeted approaches that aim to prevent or reduce mental health symptoms, such as burnout, i.e., physical and mental exhaustion, despair [12,44].

In this scoping review, it emerges that the measurement of these constructs is not uniform, as there is no consensus on which instruments are best.

The instruments used by the various studies are self-report instruments investigating the following areas: aspects of self-care, physical care, supportive relationships, mindfulness and self-compassion. The instruments help to understand a person’s capacity and self-awareness, but do not measure the person’s capacity or potential for self-care, thus suggesting an interesting area for study.

### 4.3. Limits

The main limitation of this review is that we did not specifically search for interventions targeting self-care.

The review has several additional limitations: firstly, we combined quantitative and qualitative studies in order to map documented terms and interventions; secondly, we did not use a theoretical framework in the analysis, but rather, we synthesized the information as it emerged and according to its similarity.

## 5. Conclusions

We performed a review summarizing the existing literature regarding terms related to self-care and interventions to be promoted in healthcare settings. In addition to the term self-care, we found other terms: mindfulness, compassion, and resilience. Interventions to promote self-care are divided into mindfulness-based, educational, multimodal approach, and mind-body, and describe a multifactorial process that aims to build and enhance the internal and external resources of the staff.

From our findings, however, it is evident that it is essential to improve the knowledge of the concept of self-care for health care professionals as a subject of study, preferably beginning at the training period, together with the other disciplines of the health professional’s curriculum. By identifying the factors that promote self-care and the possible negative outcomes resulting from a lack of self-care, the strategies to support individuals at a professional and personal level can be identified within organizations, with a view towards prevention, but above all, to promote well-being. Future studies may consider including (more) positive outcomes (e.g., optimism) than the usual ones (e.g., anxiety and depression).
